# The structure of SSO2064, the first representative of Pfam family PF01796, reveals a novel two-domain zinc-ribbon OB-fold architecture with a potential acyl-CoA-binding role

**DOI:** 10.1107/S1744309110002514

**Published:** 2010-03-05

**Authors:** S. Sri Krishna, L. Aravind, Constantina Bakolitsa, Jonathan Caruthers, Dennis Carlton, Mitchell D. Miller, Polat Abdubek, Tamara Astakhova, Herbert L Axelrod, Hsiu-Ju Chiu, Thomas Clayton, Marc C. Deller, Lian Duan, Julie Feuerhelm, Joanna C. Grant, Gye Won Han, Lukasz Jaroszewski, Kevin K. Jin, Heath E. Klock, Mark W. Knuth, Abhinav Kumar, David Marciano, Daniel McMullan, Andrew T. Morse, Edward Nigoghossian, Linda Okach, Ron Reyes, Christopher L. Rife, Henry van den Bedem, Dana Weekes, Qingping Xu, Keith O. Hodgson, John Wooley, Marc-André Elsliger, Ashley M. Deacon, Adam Godzik, Scott A. Lesley, Ian A. Wilson

**Affiliations:** a Joint Center for Structural Genomics, http://www.jcsg.org, USA; b Center for Research in Biological Systems, University of California, San Diego, La Jolla, California, USA; c Program on Bioinformatics and Systems Biology, Burnham Institute for Medical Research, La Jolla, California, USA; d National Institutes of Health, Bethesda, Maryland, USA; e Stanford Synchrotron Radiation Lightsource, SLAC National Accelerator Laboratory, Menlo Park, California, USA; f Department of Molecular Biology, The Scripps Research Institute, La Jolla, California, USA; g Protein Sciences Department, Genomics Institute of the Novartis Research Foundation, San Diego, California, USA; h Photon Science, SLAC National Accelerator Laboratory, Menlo Park, California, USA

**Keywords:** structural genomics, domains of unknown function, acyl-carrier proteins, acyl-coA, polyketide biosynthesis

## Abstract

SSO2064 is the first structural representative of PF01796 (DUF35), a large prokaryotic family with a wide phylogenetic distribution. The structure reveals a novel two-domain architecture comprising an N-terminal, rubredoxin-like, zinc ribbon and a C-terminal, oligonucleotide/oligosaccharide-binding (OB) fold domain. Additional N-terminal helical segments may be involved in protein–protein interactions. Domain architectures, genomic context analysis and functional evidence from certain bacterial representatives of this family suggest that these proteins form a novel fatty-acid-binding component that is involved in the biosynthesis of lipids and polyketide antibiotics and that they possibly function as acyl-CoA-binding proteins. This structure has led to a re-evaluation of the DUF35 family, which has now been split into two entries in the latest Pfam release (v.24.0).

## Abstract 2

The crystal structure of SSO2064, the first structural representative of Pfam family PF01796 (DUF35), reveals a two-domain architecture comprising an N-terminal zinc-ribbon domain and a C-terminal OB-fold domain. Analysis of the domain architecture, operon organization and bacterial orthologs combined with the structural features of SSO2064 suggests a role involving acyl-CoA binding for this family of proteins.

## 1. Introduction

In an effort to extend the structural coverage of proteins for which the biological function is unknown and cannot be deduced by homology, domain of unknown function (DUF) targets were selected from Pfam protein family PF01796 (DUF35). Here, we report the crystal structure of SSO2064, the first structural representative of this family, which was determined using the semiautomated high-throughput pipeline of the Joint Center for Structural Genomics (JCSG; http://www.jcsg.org; Lesley *et al.*, 2002 ▶) as part of the National Institute of General Medical Sciences (NIGMS) Protein Structure Initiative (PSI). The *SSO2064* gene of *Sulfolobus solfataricus*, a hyperthermoacidophilic crenarchaeon (She *et al.*, 2001 ▶), encodes a protein with a molecular weight of 16.5 kDa (residues 1–144) and a calculated isoelectric point of 6.6. Structural analysis of SSO2064 revealed two N-terminal helices followed by a rubredoxin-like zinc ribbon and an oligonucletide/oligosaccharide-binding (OB) fold domain; the genome context and operon organization suggest a role in lipid and polyketide antibiotic biosynthesis.

## 2. Materials and methods

### 2.1. Protein production and crystallization

Clones were generated using the Polymerase Incomplete Primer Extension (PIPE) cloning method (Klock *et al.*, 2008 ▶). The gene encoding SSO2064 (GenBank AAK42248; gi:13815350; Swiss-Prot Q97WQ4) was amplified by polymerase chain reaction (PCR) from *S. solfataricus* DSM 1617 (P2) genomic DNA using *PfuTurbo* DNA polymerase (Stratagene) and I-PIPE (Insert) primers (forward primer, 5′-ctgtacttccagggcATGTCTTGGGAAAAGAGTGGAAA­AGAAG-3′; reverse primer, 5′-aattaagtcgcgttaGTCAACCTTGAC­TCGTAAAGGCCACTGG-3′; target sequence in upper case) that included sequences for the predicted 5′ and 3′ ends. The expression vector pSpeedET, which encodes an amino-terminal tobacco etch virus (TEV) protease-cleavable expression and purification tag (MGSDKIHHHHHHENLYFQ/G), was PCR-amplified with V-PIPE (Vector) primers (forward primer, 5′-taacgcgacttaattaactcgtttaaacg­gtctccagc-3′; reverse primer, 5′-gccctggaagtacaggttttcgtgatgatgatgatg­atg-3′). V-PIPE and I-PIPE PCR products were mixed to anneal the amplified DNA fragments together. *Escherichia coli* GeneHogs (Invitrogen) competent cells were transformed with the V-PIPE/I-­PIPE mixture and dispensed onto selective LB–agar plates. The cloning junctions were confirmed by DNA sequencing. Expression was performed in selenomethionine-containing medium at 310 K. Selenomethionine was incorporated *via* inhibition of methionine biosynthesis (Van Duyne *et al.*, 1993 ▶), which does not require a methionine-auxotrophic strain. At the end of fermentation, lysozyme was added to the culture to a final concentration of 250 µg ml^−1^ and the cells were harvested and frozen. After one freeze–thaw cycle, the cells were sonicated in lysis buffer [50 m*M* HEPES pH 8.0, 50 m*M* NaCl, 10 m*M* imidazole, 1 m*M* tris(2-carboxyethyl)phosphine–HCl (TCEP)] and the lysate was clarified by centrifugation at 32 500*g* for 30 min. The soluble fraction was passed over nickel-chelating resin (GE Healthcare) pre-equilibrated with lysis buffer, the resin was washed with wash buffer [50 m*M* HEPES pH 8.0, 300 m*M* NaCl, 40 m*M* imidazole, 10%(*v*/*v*) glycerol, 1 m*M* TCEP] and the protein was eluted with elution buffer [20 m*M* HEPES pH 8.0, 300 m*M* imidazole, 10%(*v*/*v*) glycerol, 1 m*M* TCEP]. The eluate was buffer-exchanged with TEV buffer (20 m*M* HEPES pH 8.0, 200 m*M* NaCl, 40 m*M* imidazole, 1 m*M* TCEP) using a PD-10 column (GE Healthcare) and incubated with 1 mg of TEV protease per 15 mg of eluted protein. The protease-treated eluate was run over nickel-chelating resin (GE Healthcare) pre-equilibrated with HEPES crystallization buffer (20 m*M* HEPES pH 8.0, 200 m*M* NaCl, 40 m*M* imidazole, 1 m*M* TCEP) and the resin was washed with the same buffer. The flow-through and wash fractions were combined and concentrated to 14.5 mg ml^−1^ by centrifugal ultrafiltration (Millipore) for crystallization trials. SSO2064 was crystallized using the nanodroplet vapor-diffusion method (Santarsiero *et al.*, 2002 ▶) with standard JCSG crystallization protocols (Lesley *et al.*, 2002 ▶). Sitting drops composed of 200 nl protein mixed with 200 nl crystallization solution were equilibrated against 50 µl reservoir at 277 K for 10 d prior to harvesting. The crystallization reagent consisted of 2.0 *M* ammonium sulfate and 0.1 *M* acetate pH 4.6. Glycerol was added to the crystal as a cryoprotectant to a final concentration of 15%(*v*/*v*). Initial screening for diffraction was carried out using the Stanford Automated Mounting system (SAM; Cohen *et al.*, 2002 ▶) at the Stanford Synchrotron Radiation Lightsource (SSRL, Menlo Park, California, USA). The diffraction data were indexed in the tetragonal space group *P*4_1_22. The oligomeric state of SSO2064 was determined using a 0.8 × 30 cm Shodex Protein KW-803 column (Thomson Instruments) equilibrated in 20 m*M* Tris, 200 m*M* NaCl, 0.5 m*M* TCEP pH 7.5 and pre-calibrated with gel-filtration standards (Bio-Rad).

### 2.2. Data collection, structure solution and refinement

Multiple-wavelength anomalous diffraction (MAD) data were collected on beamline BL11-1 at the SSRL at wavelengths corresponding to the inflection (λ_2_), high-energy remote (λ_1_) and peak (λ_3_) of a selenium MAD experiment. The data sets were collected at 100 K with an ADSC Q315 CCD detector using the *Blu-Ice* data-collection environment (McPhillips *et al.*, 2002 ▶). The MAD data were integrated and reduced using *XDS* and scaled with the program *XSCALE* (Kabsch, 1993 ▶). The heavy-atom sites were determined with *SHELXD* (Sheldrick, 2008 ▶) and phasing was performed with *autoSHARP* (Bricogne *et al.*, 2003 ▶; mean figure of merit of 0.23 with five sites). Automated model building was performed with *ARP*/*wARP* (Cohen *et al.*, 2004 ▶). X-ray fluorescence excitation scans showed peaks consistent with the *K*-shell emission lines of selenium and zinc. No peaks were observed for other metals. Furthermore, X-­ray fluorescence wavelength scans around the zinc and selenium *K* absorption edges showed clear transitions. After the model had been built, *autoSHARP* was run with the correct heavy-atom element assignment. The final heavy-atom model contained two selenium sites (corresponding to SeMet19 from both chains) and two zinc sites and resulted in an improved mean figure of merit (0.27). Model com­pletion and refinement were performed with *Coot* (Emsley & Cowtan, 2004 ▶) and *REFMAC*5 (Winn *et al.*, 2003 ▶) using data set λ_1_. The refinement included experimental phase restraints in the form of Hendrickson–Lattman coefficients from *SHARP* and TLS refinement with one TLS group per chain. The refined *B* values for the Zn atoms are slightly lower than those of the coordinating S atoms and support modeling the zinc sites as fully occupied. Together with the X-ray fluorescence data, this suggests that endogenous zinc co-purified with the protein and was not exchanged on the nickel column. Data-reduction and refinement statistics are summarized in Table 1 ▶.

### 2.3. Validation and deposition

**Figure 1:**
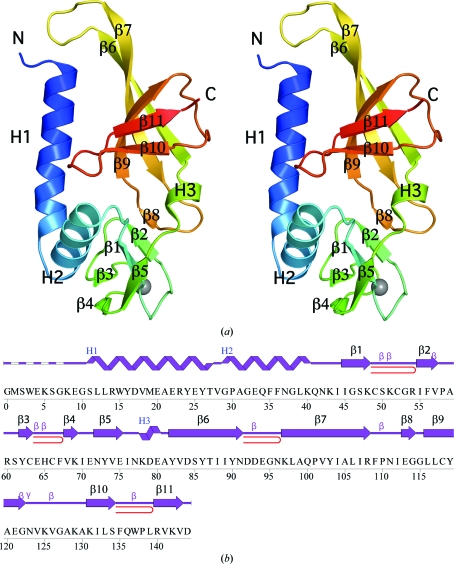
Crystal structure of SSO2064 from *S. solfataricus*. (*a*) Stereo ribbon diagram of the SSO2064 monomer (chain *A*) color-coded from the N-terminus (blue) to the C-terminus (red). Helices (H1–H3) and β-strands (β1–β11) are indicated. The zinc ion is depicted as a gray sphere. (*b*) Diagram showing the secondary-structure elements of SSO2064 superimposed on its primary sequence. The labeling of secondary-structure elements is in accord with *PDBsum* (http://www.ebi.ac.uk/pdbsum): α-helices are labeled H1 and H2, the 3_10_-helix is labeled H3, the β-strands are labeled β1–β11, β-turns and γ-turns are designated by their respective Greek letters (β, γ) and red loops indicate β-hairpins.

**Figure 2:**
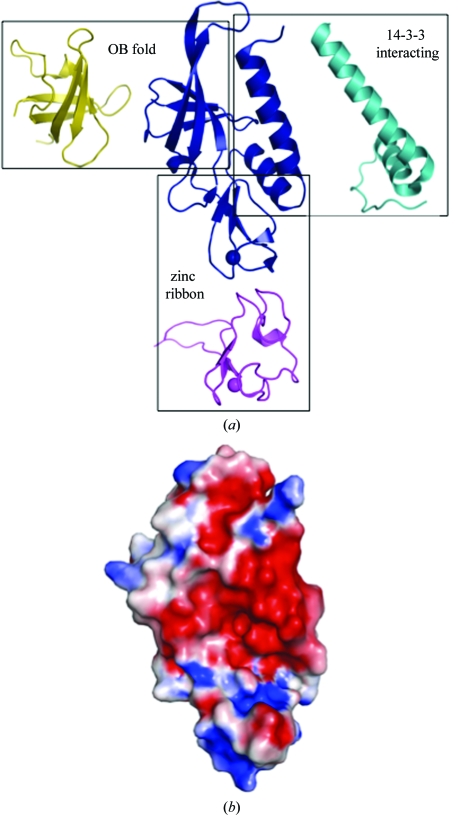
SSO2064 exhibits structural similarity to protein–protein interaction motifs, rubredoxin-like zinc ribbons and OB folds. (*a*) Ribbon diagram showing the structural superposition of SSO2064 (PDB code 3irb; residues 8–144; blue) with the 14-3-3 interacting motif of plant H^+^-ATPase (PDB code 2o98; residues 7–56; cyan), a zinc-substituted rubredoxin from *Guillardia theta* (PDB code 1h7v; residues 1–60; magenta) and the major cold-shock protein from *E. coli* (PDB code 1mjc; residues 1–70; yellow). Superposed proteins have been translated for clarity. Zinc ions are indicated as spheres. (*b*) Electrostatic surface representation of SSO2064 in the same orientation as in (*a*). Positive potential is in blue (+7*kT*e^−1^) and negative potential is in red (−7*kT*e^−1^).

The quality of the crystal structure was analyzed using the *JCSG Quality Control* server (http://smb.slac.stanford.edu/jcsg/QC). This server processes the coordinates and data using a variety of validation tools including *AutoDepInputTool* (Yang *et al.*, 2004 ▶), *MolProbity* (Davis *et al.*, 2007 ▶), *WHAT IF* 5.0 (Vriend, 1990 ▶), *RESOLVE* (Terwilliger, 2003 ▶) and *MOLEMAN*2 (Kleywegt, 2000 ▶) as well as several in-house scripts and summarizes the output. Protein quaternary-structure analysis used the *PISA* server (Krissinel & Henrick, 2007 ▶). Fig. 1 ▶(*b*) was adapted from an analysis using *PDBsum* (Laskowski *et al.*, 2005 ▶) and all other figures were prepared using *PyMOL* (DeLano Scientific). Invariant regions between the two chains were calculated using *ESCET* (Schneider, 2002 ▶). Fig. 2 ▶(*b*) was prepared using the *PDB*2*PQR* server (Dolinsky *et al.*, 2007 ▶) and the *APBS* module (Dolinsky *et al.*, 2007 ▶; Baker *et al.*, 2001 ▶) in *PyMOL* with default parameters.

Atomic coordinates and experimental structure factors for SSO2064 at 1.80 Å resolution have been deposited in the PDB (http://www.wwPDB.org) and are accessible under the code 3irb.

## 3. Results and discussion

### 3.1. Protein structure description

The crystal structure of SSO2064 (Fig. 1 ▶ *a*) was determined to 1.80 Å resolution using the MAD phasing technique. Data-collection, model and refinement statistics are summarized in Table 1 ▶. The final model included residues 8–144 for chain *A*, residues 10–27 and 29–144 for chain *B*, two acetate molecules, six sulfate ions, two zinc ions and 201 water molecules in the asymmetric unit (ASU). No electron density was observed for the N-terminal glycine (Gly0) which remained after cleavage of the expression and purification tag, for the first seven residues of chains *A* and *B* and for Lys8, Glu9 and Val28 in chain *B*. The side-chain atoms of Lys8, Glu33, Lys40, Lys69, Lys97, Lys129 and Lys131 in chain *A* and Glu33, Lys40, Lys69, Lys97, Lys125, Lys129 and Lys131 in chain *B* were omitted owing to weak or absent electron density. The Matthews coefficient (*V*_M_; Matthews, 1968 ▶) is 2.4 Å^3^ Da^−1^ and the estimated solvent content is 49.8%. The Ramachandran plot produced by *MolProbity* (Davis *et al.*, 2007 ▶) indicated that 100% of the residues are in favored regions. Pro58 and Pro138 are in the *cis*-­conformation in both chains and are supported by clear and un­ambiguous electron density.

### 3.2. Overall structure

The entire amino-acid sequence of SSO2064 was originally classified as part of the DUF35 family. However, the structure clearly revealed a two-domain organization (Fig. 1 ▶ *a*). *SCOP* (v.1.75) classifies SSO2064 as an OB fold (residues 77–144) containing an N-terminal zinc-ribbon subdomain (residues 43–76). In the crystal structure, the zinc is coordinated by Cys49, Cys52, Cys63 and Cys66 of the rubredoxin-like, zinc-ribbon fold (http://scop.mrc-lmb.cam.ac.uk/scop/data/scop.b.c.hb.h.bf.b.html). Two N-terminal helices preceding the zinc-ribbon domain complete the structure and are involved in crystal-packing interactions. The two-domain architecture of SSO2064 is conserved in all DUF35 homologs. This structure has led to a re-evaluation of the Pfam DUF35 family which, as a result of our study, has been split into two entries in the latest Pfam release (Pfam 24.0, October 2009). The original DUF35 entry has been truncated and now represents the OB-fold domain. A new entry, DUF35_N (PF12172), has been created to represent the rubredoxin-like zinc-ribbon domain.

The two molecules in th ASU are similar, with an overall C^α^ r.m.s.d. of 1.4 Å over 137 residues. The main differences are localized in the regions between helices H1 and H2, strands β3–β4 and strands β6–β7, which display different conformations (Supplementary Fig. S1^1^). None of these regions are involved in crystal contacts, suggesting possible functional relevance of these conformationally flexible regions. Excluding these regions, the core of the structure can be superimposed with a C^α^ r.m.s.d. of 0.45 Å over 97 residues. Chain *A* was used in all subsequent analyses as it contained fewer disordered residues.

As expected, a search with *FATCAT* (Ye & Godzik, 2004 ▶) revealed similarities to both OB and rubredoxin-like folds (Fig. 2 ▶). The most similar OB folds were those belonging to DNA-interacting proteins, including replication factors [PDB codes 3dm3 (J. Seetharaman, M. Su, M. Maglaqui, H. Janjua, C. Ciccosanti, R. Xiao, R. Nair, J. K. Everett, T. B. Acton, B., Rost, G. T. Montelione, L. Tong & J. F. Hunt, unpublished; r.m.s.d. of 1.9 Å over 76 residues; 11% sequence identity) and 2pi2 (Deng *et al.*, 2007 ▶; r.m.s.d. of 1.3 Å over 74 residues; 5% sequence identity)] and transcription factors, such as Y-box-binding proteins (PDB code 1h95; Kloks *et al.*, 2002 ▶; r.m.s.d. of 2.0 Å over 78 residues; 0% sequence identity), polymixin-resistance protein (PDB code 2jso; Fu *et al.*, 2007 ▶; r.m.s.d. of 3.1 Å over 65 residues; 11% sequence identity) and major cold-shock protein (PDB code 1mjc; Schindelin *et al.*, 1994 ▶; r.m.s.d. of 2.4 Å over 74 residues; 5% sequence identity), although in all cases sequence identity was <15%. The two N-terminal helices showed the greatest similarity to the 14-3-3 interacting region of a plant H^+^-ATPase (PDB code 2o98; Ottmann *et al.*, 2007 ▶; r.m.s.d. of 3.1 Å over 45 residues), although again the sequence identity was low at only 5%.

A search with *PISA* (Krissinel & Henrick, 2007 ▶) suggested that the likely quaternary structure of SSO2064 was either a tetramer or a dimer. In both cases consecutive monomers are oriented antiparallel to one another with the N-terminal helices packing at the center and the zinc ribbons on the outside. The tetramer buries a total of ∼3800 Å^2^, including 32 hydrogen bonds and four salt bridges, while the dimer buries a surface area of 1050 Å^2^. However, the N-terminal segment of helix H1, which is substantially involved in both dimer and tetramer formation, is absent in many homologs, suggesting that this oligomerization state may only be encountered in a subset of the DUF35 family or is of limited functional relevance. However, analytical size-exclusion chromatography in combination with static light scattering strongly suggests that a monomer is likely to be the biologically relevant state of the molecule.

Zinc ribbons, a structurally distinct group of zinc fingers, are short (typically 20–50 residues), zinc-stabilized structural motifs that play a diverse set of functional roles, serving as modules that bind nucleic acids, proteins and small molecules (Krishna *et al.*, 2003 ▶). OB folds are small, five-stranded, mixed β-barrels connected by loops that modulate ligand binding. Their ligands include oligosaccharides, oligo­nucleotides, proteins, metal ions and catalytic substrates. An electrostatic surface representation of the SSO2064 monomer shows a groove formed along the OB and zinc-ribbon-like folds with a hydrophobic and a basic potential, respectively (Fig. 2 ▶ *b*). Some of the most highly conserved residues among homologs (Pro58, Arg60, Ser82 and Thr87) line this groove, suggesting that it might serve as a binding site.

Analysis of the OB-fold architecture (Arcus, 2002 ▶) has shown that the binding face is consistently centered along the second and third strands of the OB-fold β-barrel and is bordered by loops β2–β3 and β3–β4 at the two ends of the barrel. In SSO2064 these, regions correspond to strands β7 and β9 and loops β6–β7 and β7–β9 (which includes the small β8 strand). The outermost edge of loop β6–β7 contains an acidic sequence (Asp92, Asp93, Glu94), with the side-chain carboxyl of Asp92 maintaining the loop conformation through interactions with the main-chain N atoms of Glu94 and Asn96. Asp93 forms hydrogen bonds with the amide N atoms at the beginning of helix H1 and Glu94 being likely to interact with basic side chains (*e.g.* Lys8). These interactions may serve to maintain this helix in position with respect to both the OB fold and the interfacing monomer in the dimer although, as discussed earlier, the variable length of helix H1 indicates that the conformation of loop β6–β7 is also likely to vary. The variable length of helix H1 and the different conformations of the β6–β7 loop in the two chains of SSO2064 (Supplementary Fig. S1^1^) lend support to this hypothesis. Similarly, the conformation of the long β7–β9 loop is maintained in part *via* the short β8 strand hydrogen bonding to part of the zinc ribbon (Fig. 1 ▶ *b*). While loop β7–β9 directly forms part of the groove (Fig. 2 ▶ *b*), loop β6–β7 does not as it is sterically hindered by helix H1. However, in homologs with a shorter helix H1, the β6–β7 loop would no longer be occluded and could also possibly form part of this binding site.

### 3.3. Comparison with other OB-fold-containing and zinc-ribbon-containing proteins

**Figure 3:**
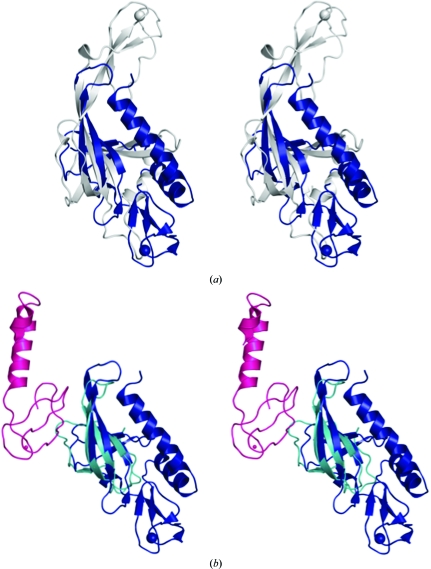
SSO2064 subdomains display a novel spatial organization with respect to other OB-fold-containing and zinc-ribbon-containing proteins. Stereo ribbon diagram showing the structural superposition of SSO2064 (PDB code 3irb; residues 8–144; blue) with (*a*) the mini-chromosome maintenance complex (MCM) from *M. thermoautotrophicum* (PDB code 1ltl; residues 95–242; gray) and (*b*) chains *A* (residues 28–83; cyan) and *Y* (residues 10–71; magenta) from the large ribosomal subunit from *H. marismortui* (PDB code 1jj2; Klein *et al.*, 2001 ▶). Zinc (gray and blue) and cobalt (magenta) [chain *Y* in (*b*)] are indicated as spheres.

Several structures of proteins containing OB folds and zinc ribbons have previously been described. The structure of a fragment of the mini-chromosome maintenance (MCM) protein from *Methanobacterium thermoautotrophicum* (Fig. 3 ▶ *a*) revealed that the zinc ribbon inserted within the OB-fold loops is implicated in higher order oligomerization, while the OB barrel itself is involved in interactions with DNA (Fletcher *et al.*, 2003 ▶). In replication protein A (RPA), a eukaryotic DNA-binding protein, the OB fold is involved in ss-DNA binding, while the zinc ribbon is suggested to contribute to cooperativity or regulate DNA binding *via* redox effects (Bochkareva *et al.*, 2002 ▶). Both the zinc ribbon and OB fold are involved in RPA trimerization, forming a proteolytically resistant core. Associations of OB folds with zinc motifs are also encountered between separate polypeptide chains. The structure of the *Haloarcula marismortui* large ribosomal subunit shows that the rubredoxin-like zinc ribbon (protein L37Ae) interacts extensively with RNA (Klein *et al.*, 2001 ▶), as well as with the OB fold of the N-terminal domain of ribosomal protein L2 (Fig. 3 ▶ *b*). The latter is also involved in RNA binding.

To our knowledge, all combinations of OB folds and zinc ribbons, whether intramolecular or intermolecular, involve nucleic acid-binding proteins. In all cases, the zinc ribbon is located on the opposite side of the OB barrel with respect to SSO2064 (Figs. 3 ▶ *a* and 3 ▶ *b*). The OB fold and the zinc ribbon are often implicated in oligomerization.

### 3.4. Genome-context analysis

SSO2064 homologs are fused to a variety of other domains, such as (i) members of the thiolase superfamily, (ii) NAD(P)-binding Rossmann-fold domains related to the short-chain acyl-CoA dehydro­genases, (iii) the sterol-carrier protein family (SCP2) and (iv) dehydratases of the hot-dog superfamily (Dillon & Bateman, 2004 ▶). Further genome-context analysis (http://string.embl.de) indicated that the members of this family show a strong gene-neighborhood association with members of the thiolase superfamily (EC 2.3.1.9) that are involved in condensation of acyl-CoA moieties in the formation of longer chain aliphatic and cyclic skeletons. Importantly, an operon that combines genes encoding an ortholog of SSO2064 and an active, as well as an inactive, member of the thiolase superfamily is found in *Pseudomonas fluorescens* (the *phlABC* operon, where *phlB* is an ortholog of *SSO2064*). The products of this operon, together with the polyketide synthase PhlD, are required for the biosynthesis of the polyketide antibiotic 2,4-diacetylphloroglucinol (2,4-DAPG; Bangera & Thomashow, 1999 ▶). The three-protein complex formed by the *phlABC* operon is absolutely required to catalyze the condensation of two acetyl-CoA molecules to form acetoacetyl-CoA in the first step of 2,4-DAPG biosynthesis. These *phlABC*-encoded proteins are again required in the final step to convert monoacetylphloro­glucinol to 2,4-DAPG by adding an acetyl group. In this complex, PhlC is the catalytically active thiolase-superfamily protein that catalyzes the condensation of the acetyl-CoA molecules. PhlA is an inactive member of the thiolase superfamily that is likely to regulate the length of the poly-β-ketone (Hutchinson & Fujii, 1995 ▶), as in other bacterial polyketide-biosynthesis pathways. Given that the SSO2064 ortholog PhlB, like other members of this family, lacks a conservation pattern suggestive of an enzymatic role, it is most likely functions as the acyl-CoA carrier protein in the reaction. Consistent with the other domain architectures described above, it is likely that members of this family, including SSO2064, function as noncovalent acyl-CoA-delivery proteins in different acyl-CoA-utilizing reactions. The presence of certain members of the family with two tandemly repeated modules (each corresponding to a ‘DUF35’ unit), as well as their oligomeric structures, suggest that each module probably interacts with a single acyl-CoA unit. While several examples of OB-fold domains bind low-molecular-weight compounds (Anantharaman *et al.*, 2001 ▶), to our knowledge, this family appears to represent the first instance of an OB fold adapted to bind an acyl-CoA moiety.

In structural terms, the zinc ribbon of SSO2064 is closest to the zinc ribbons of nucleic acid-binding proteins, such as the reverse gyrase and DNA-replication primosomal proteins. Otherwise, the OB-fold domain does not show a particularly close relationship to other small-molecule-binding OB-fold domains. Thus, these domains may have been independently adapted apparently for small-molecule binding in the SSO2064 family. We speculate that the N-terminal zinc ribbon contacts the nucleotide moiety of acyl-CoA in a manner similar to that seen in nucleic acid-binding zinc ribbons, while the hydrophobic surface of the OB fold (formed by strand β6 and loop β7–β9) could accommodate the acyl side chain.

The SSO2064 protein family [DUF35 (PF01796)] contains around 650 homologs from both archaea and bacteria, including several pathogens, such as mycobacteria, burkholderia, firmicutes and spiro­chaetes. However, we have thus far not detected any member of this family in eukaryotes. Based on this phyletic pattern, their predicted small-molecule binding function and their presence in pathogenic bacteria, we propose that members of this family could serve as potential targets for therapeutic intervention. In addition, their role in polyketide-antibiotic biosynthesis suggests that members of this family could be used for engineering pathways for generating such biomedically important compounds. We, therefore, expect that the structure presented here should inspire further biochemical and biophysical studies on this novel family of protein implicated in lipid and polyketide biosynthesis. Models of SSO2064-family proteins can be accessed at http://www1.jcsg.org/cgi-bin/models/get_mor.pl?key=3irbA.

Additional information about SSO2064 is available from *TOPSAN* (Krishna *et al.*, 2010 ▶) at http://www.topsan.org/explore?PDBid=3irb. A list of all members of the DUF35 family that have been worked on by structural genomics centers is available *via* TargetDB (Chen *et al.*, 2004 ▶) of the PSI-Knowledgebase (Berman *et al.*, 2009 ▶) at http://targetdb.pdb.org/servlet/TargetSearch?which_seq=SG&format=html&pdbid=PF01796&cp=1.

## 4. Conclusions

The first representative of PF01796 reveals a rubredoxin-like, zinc ribbon and an OB fold in a novel arrangement that are likely to cooperate to bind an acyl-CoA moiety.

## Supplementary Material

PDB reference: SSO2064 from *S. solfataricus*, 3irb

Supplementary material file. DOI: 10.1107/S1744309110002514/wd5125sup1.pdf

## Figures and Tables

**Table 1:** Summary of crystal parameters and data-collection and refinement statistics for SSO2064 (PDB code 3irb) Values in parentheses are for the highest resolution shell.

	λ_1_ MADSe	λ_2_ MADSe	λ_3_ MADSe
Data collection			
Space group	*P*4_1_22		
Unit-cell parameters (Å)	*a* = *b* = 75.17, *c* = 115.40		
Wavelength (Å)	0.9184	0.9794	0.9792
Resolution range (Å)	26.9–1.80 (1.85–1.80)	26.9–1.80 (1.85–1.80)	29.1–1.91 (1.91–1.96)
No. of observations	221293	220707	170456
No. of unique reflections	31362	31365	26484
Completeness (%)	99.8 (99.3)	99.8 (99.3)	99.8 (99.9)
Mean *I*/σ(*I*)	13.2 (2.6)	13.0 (2.6)	12.0 (2.0)
*R*_merge_† on *I* (%)	8.8 (87.3)	8.8 (87.2)	12.9 (117.7)
*R*_meas_‡ on *I* (%)	9.5 (94.2)	9.5 (94.1)	14.0 (127.7)
Model and refinement statistics			
Resolution range (Å)	26.9–1.80		
No. of reflections (total)	31317§		
No. of reflections (test)	1560		
Completeness (%)	99.8		
Data set used in refinement	λ_1_ MADSe		
Cutoff criterion	|*F*| > 0		
*R*_cryst_¶	0.169		
*R*_free_††	0.200		
Stereochemical parameters			
Restraints (r.m.s.d. observed)			
Bond angles (°)	1.41		
Bond lengths (Å)	0.014		
Average isotropic *B* value (Å^2^)	29.4		
ESU‡‡ based on *R*_free_	0.105		
No. of protein residues/atoms	271/2187		
No. of water/other solvent molecules and ions	201/10§§		

† *R*_merge_ = 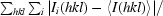 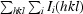.

‡ *R*_meas_ = 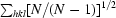 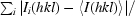 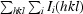 (Diederichs & Karplus, 1997 ▶).

§ Typically, the number of unique reflections used in refinement is slightly less than the total number that were integrated and scaled. Reflections are excluded owing to systematic absences, negative intensities and rounding errors in the resolution limits and unit-cell parameters.

¶ *R*_cryst_ = 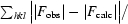 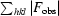, where *F*_calc_ and *F*_obs_ are the calculated and observed structure-factor amplitudes, respectively.

†† *R*_free_ is the same as *R*_cryst_, but for 5.0% of the total reflections chosen at random and omitted from refinement.

‡‡ Estimated overall coordinate error (Collaborative Computational Project, Number 4, 1994 ▶; Cruickshank, 1999 ▶).

§§ Two Zn ions, two acetates and six sulfates.

## Footnotes

1 Supplementary material has been deposited in the IUCr electronic archive (Reference: WD5125).
