# Supplementary material for: The structure of SSO2064, the first representative of Pfam family PF01796, reveals a novel two-domain zinc-ribbon OB-fold architecture with a potential acyl-CoA-binding role
Source: Acta Crystallogr Sect F Struct Biol Cryst Commun. 2010 Mar 5;66(Pt 10):1160–6. doi: 10.1107/S1744309110002514 (PMC2954200; doi:10.1107/S1744309110002514)
Supplement: Supplementary file 1 [file f-66-01160-sup1.pdf]

Supplementary Material

Figure S1. Comparison of the SSO2064 monomers. (A) Stereo ribbon diagram showing a C^{α} superposition of SSO2064 chains A and B (PDB id: 3irb). Invariant regions are in blue, variable ones in red. **(B)** Error-scaled pair-wise difference distance matrix plotted from 5σ to 10σ for the two SSO2064 chains. Color intensities indicate the magnitude of distance change with a scale bar given below.

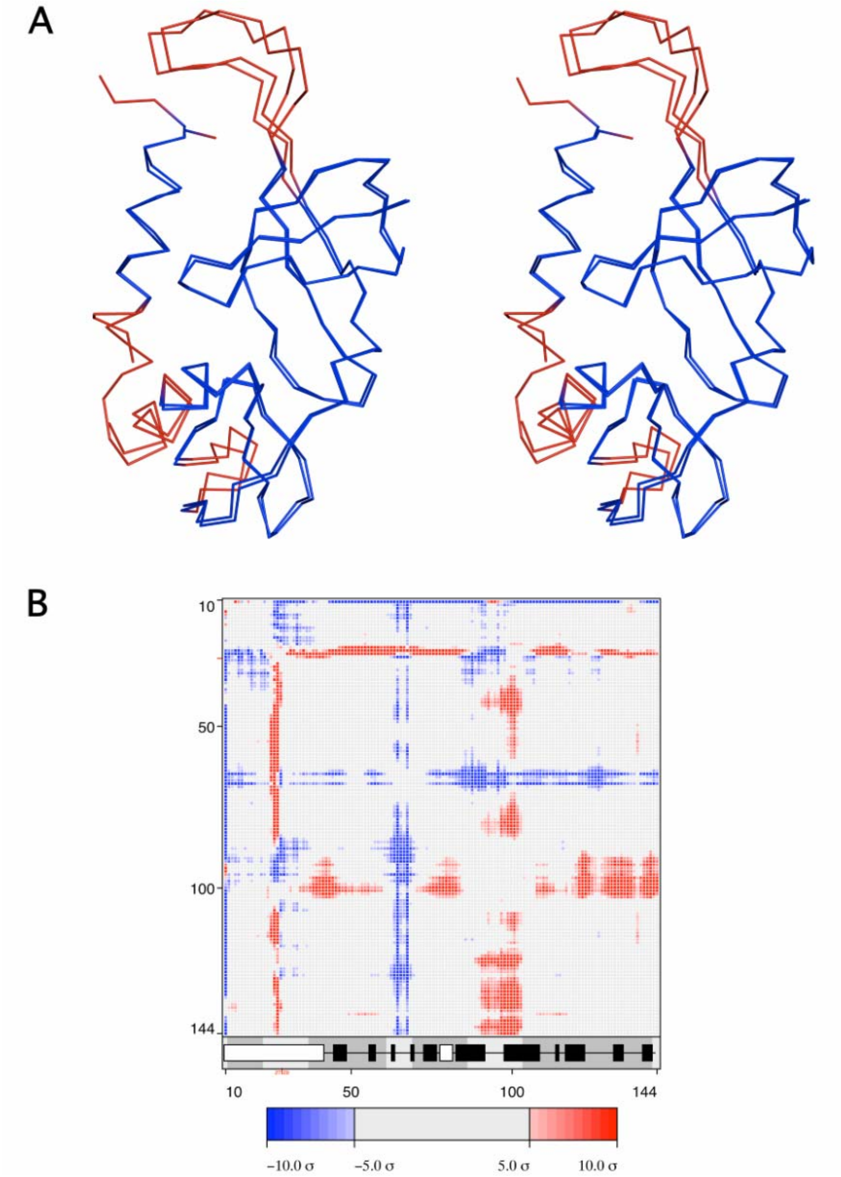

FIGURE S1
